# ﻿GPS tracking data of Eurasian oystercatchers (*Haematopusostralegus*) from the Netherlands and Belgium

**DOI:** 10.3897/zookeys.1123.90623

**Published:** 2022-10-03

**Authors:** Henk-Jan van der Kolk, Peter Desmet, Kees Oosterbeek, Andrew M. Allen, Martin J. Baptist, Roeland A. Bom, Sarah C. Davidson, Jan de Jong, Hans de Kroon, Bert Dijkstra, Rinus Dillerop, Adriaan M. Dokter, Magali Frauendorf, Tanja Milotić, Eldar Rakhimberdiev, Judy Shamoun-Baranes, Geert Spanoghe, Martijn van de Pol, Gunther Van Ryckegem, Joost Vanoverbeke, Eelke Jongejans, Bruno J. Ens

**Affiliations:** 1 Netherlands Institute of Ecology, Department of Animal Ecology, Wageningen, Netherlands Centre of Avian Population Studies (CAPS) Wageningen Netherlands; 2 Dutch Bryological and Lichenological Society (BLWG), Utrecht, Netherlands Netherlands Institute of Ecology, Department of Animal Ecology Wageningen Netherlands; 3 Radboud University, Nijmegen, Netherlands Radboud University Nijmegen Netherlands; 4 Centre of Avian Population Studies (CAPS), Wageningen, Netherlands Dutch Bryological and Lichenological Society (BLWG) Utrecht Netherlands; 5 Research Institute for Nature and Forest (INBO), Brussels, Belgium Research Institute for Nature and Forest (INBO) Brussels Belgium; 6 Sovon Dutch Centre for Field Ornithology, Nijmegen, Netherlands Sovon Dutch Centre for Field Ornithology Nijmegen Netherlands; 7 Wageningen Marine Research, Wageningen University and Research, Den Helder, Netherlands Wageningen University and Research Den Helder Netherlands; 8 Royal Netherlands Institute for Sea Research, Coastal Systems, ‘t Horntje, Netherlands Royal Netherlands Institute for Sea Research 't Horntje Netherlands; 9 Department of Animal Migration, Max Plank Institute of Animal Behaviour, Radolfzell, Germany Department of Animal Migration, Max Plank Institute of Animal Behaviour Radolfzell Germany; 10 WetlandWacht, Vogelbescherming, Zeist, Netherlands WetlandWacht, Vogelbescherming Zeist Netherlands; 11 Vogelwerkgroep Assen, Assen, Netherlands Vogelwerkgroep Assen Assen Netherlands; 12 Cornell Lab of Ornithology, Cornell University, Ithaca, USA Cornell University Ithaca United States of America; 13 Institute for Biodiversity and Ecosystem Dynamics, University of Amsterdam, Amsterdam, Netherlands University of Amsterdam Amsterdam Netherlands; 14 College of Science and Engineering, James Cook University, Townsville, Australia James Cook University Townsville Australia

**Keywords:** Acceleration measurements, animal movement, behaviour, bio-logging, bird tracking, habitat use, machine observation, Movebank, oystercatchers, time budget, UvA-BiTS

## Abstract

We describe six datasets that contain GPS and accelerometer data of 202 Eurasian oystercatchers (*Haematopusostralegus*) spanning the period 2008–2021. Birds were equipped with GPS trackers in breeding and wintering areas in the Netherlands and Belgium. We used GPS trackers from the University of Amsterdam Bird Tracking System (UvA-BiTS) for several study purposes, including the study of space use during the breeding season, habitat use and foraging behaviour in the winter season, and impacts of human disturbance. To enable broader usage, all data have now been made open access. Combined, the datasets contain 6.0 million GPS positions, 164 million acceleration measurements and 7.0 million classified behaviour events (i.e., flying, walking, foraging, preening, and inactive). The datasets are deposited on the research repository Zenodo, but are also accessible on Movebank and as down-sampled occurrence datasets on the Global Biodiversity Information Facility (GBIF) and Ocean Biodiversity Information System (OBIS).

## ﻿Described datasets

Oosterbeek K, Bom RA, Shamoun-Baranes J, Desmet P, van der Kolk H, Bouten W, Ens BJ (2022) O_SCHIERMONNIKOOG - Eurasian oystercatchers (*Haematopusostralegus*, Haematopodidae) breeding on Schiermonnikoog (the Netherlands). Dataset. https://doi.org/10.5281/zenodo.6603183

Oosterbeek K, de Jong J, Desmet P, van der Kolk H, Bouten W, Ens BJ (2022) O_AMELAND - Eurasian oystercatchers (*Haematopusostralegus*, Haematopodidae) breeding on Ameland (the Netherlands). Dataset. https://doi.org/10.5281/zenodo.6656937

Dokter AM, Oosterbeek K, Baptist M, Desmet P, van der Kolk H, Bouten W, Ens BJ (2022) O_BALGZAND - Eurasian oystercatchers (*Haematopusostralegus*, Haematopodidae) wintering on Balgzand (the Netherlands). Dataset. https://doi.org/10.5281/zenodo.6603023

van der Kolk H, Oosterbeek K, Jongejans E, Frauendorf M, Allen AM, Bouten W, Desmet P, de Kroon H, Ens BJ, van de Pol M (2022) O_VLIELAND - Eurasian oystercatchers (*Haematopusostralegus*, Haematopodidae) breeding and wintering on Vlieland (the Netherlands). Dataset. https://doi.org/10.5281/zenodo.5653891

Dijkstra B, Dillerop R, Oosterbeek K, Bouten W, Desmet P, van der Kolk H, Ens BJ (2022) O_ASSEN - Eurasian oystercatchers (*Haematopusostralegus*, Haematopodidae) breeding in Assen (the Netherlands). Dataset. https://doi.org/10.5281/zenodo.5653311

Spanoghe G, Desmet P, Milotic T, Van Ryckegem G, Vanoverbeke J, Ens BJ, Bouten W (2022) O_WESTERSCHELDE - Eurasian oystercatchers (*Haematopusostralegus*, Haematopodidae) breeding in East Flanders (Belgium). Dataset. https://doi.org/10.5281/zenodo.5879096

## ﻿Introduction

The nominate subspecies of the Eurasian oystercatcher (*Haematopusostralegusostralegus* Linnaeus, 1758) is a well-studied, long-lived wader that breeds in coastal areas, and locally inland, in large parts of Europe and winters in coastal areas in Europe and northern Africa (van de Pol et al. 2014). In coastal areas, oystercatchers largely rely on intertidal mudflats where they forage on shellfish and worms. The behavioural ecology and population ecology of oystercatcher are well understood, as showcased by numerous studies on individual variation in dominance, foraging techniques (Goss-Custard 1996) and life history (Ens et al. 2014a), long-term population studies (Allen et al. 2022) and development of models that predict winter mortality from individual-based models (Stillman and Goss-Custard 2010). The Netherlands harbours approximately 10% of the global breeding population and 20% of the wintering population of the Eurasian oystercatcher, whereas Belgium harbours a small part of the breeding and winter population (~0.1%; van de Pol et al. 2014). The population of oystercatchers increased during the second half of the 20^th^ century, stabilized in the 1980’s, but afterwards declined strongly (van de Pol et al. 2014). There is an increasing concern about the ongoing decline in the Netherlands, for which potential causes include (mechanical and non-mechanical) fisheries, disturbance, agricultural intensification and rising sea levels due to climate change (van de Pol et al. 2014).

The datasets described here include all GPS tracking efforts of Eurasian oystercatchers in the Netherlands and Belgium. Research on oystercatchers in the Netherlands intensified in 2008, which was declared as the “Year of the Oystercatcher” by BirdLife Netherlands and the Sovon Dutch Centre for Field Ornithology. In that year, ringing groups were established through the country and they started to colour-band oystercatchers at their breeding grounds, such that they could be resighted in the wintering areas. In the same year, the first trials were completed using GPS trackers from the University of Amsterdam Bird Tracking System (UvA-BiTS; Bouten et al. 2013) on oystercatchers on Schiermonnikoog, an island in the Wadden Sea where a breeding population of oystercatchers has been monitored since 1983. In 2010, new UvA-BiTS tracking studies on oystercatchers began on the Wadden island of Ameland and in the tidal basin of Balgzand. In 2016, the CHIRP (Cumulative Human Impact on biRd Populations) project started (Allen et al. 2018), which aimed to quantify the cumulative impact of human activities on the oystercatcher population. Within this project oystercatchers were equipped with UvA-BiTS GPS trackers on the Wadden island of Vlieland. In 2018, two smaller UvA-BiTS GPS tracking projects were initiated in the city of Assen (Drenthe, the Netherlands) and in agricultural areas near the city of Antwerp (Belgium, in close proximity to the estuary of the Scheldt River).

The research objectives of the GPS tracking studies presented here were diverse, and included studying the territory size and territory use of breeding oystercatchers on saltmarshes and roof-nesting birds in cities, studying the spatial use of mudflats in winter with regard to the presence of benthic prey and to quantify the impacts of aircraft disturbance. To enable further use of the tracking data, we have now published all of the collected data as open data under Creative Commons Zero (CC0 1.0) waiver.

## ﻿Coverage

### ﻿Taxonomic coverage

**Figure 1.**
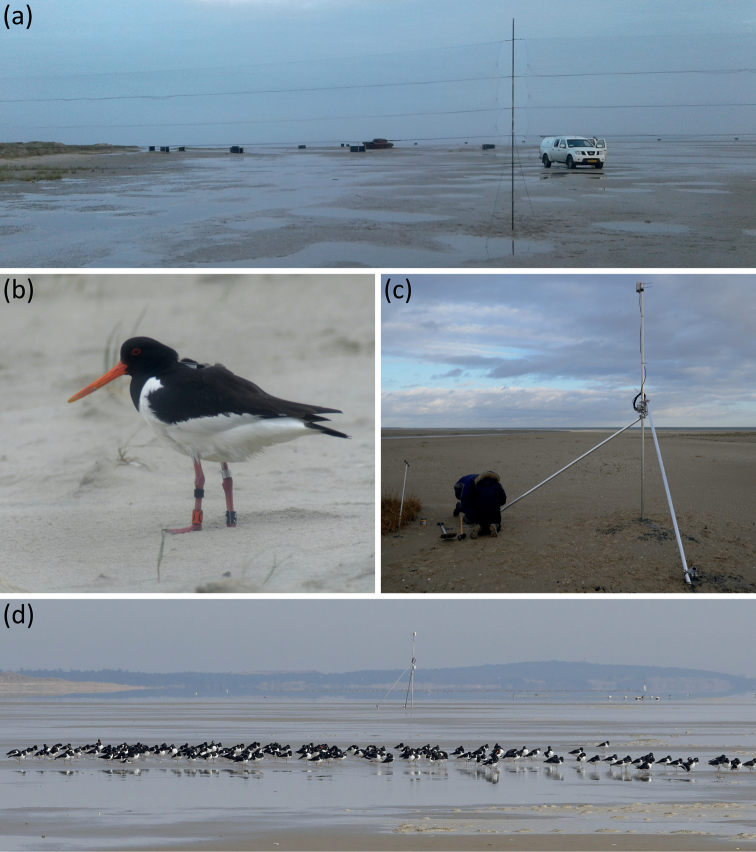
Collecting GPS data of Eurasian oystercatchers using the UvA-BiTS system. **a** mistnets at a high tide roost site on Vlieland, with which birds were trapped **b** Eurasian oystercatcher equipped with colour-rings and UvA-BiTS GPS tracker **c** installation of a relay station via which data from trackers could be retrieved **d** a high tide roost site of Eurasian oystercatchers, showing a relay station in the background.

The six datasets collectively contain 6.0 million GPS locations and 164 million accelerometer measurements of 202 individuals of the nominate subspecies of the Eurasian oystercatcher *Haematopusostralegusostralegus*, collected using UvA-BiTS (Fig. 1).

### ﻿Geographic coverage

**Figure 2.**
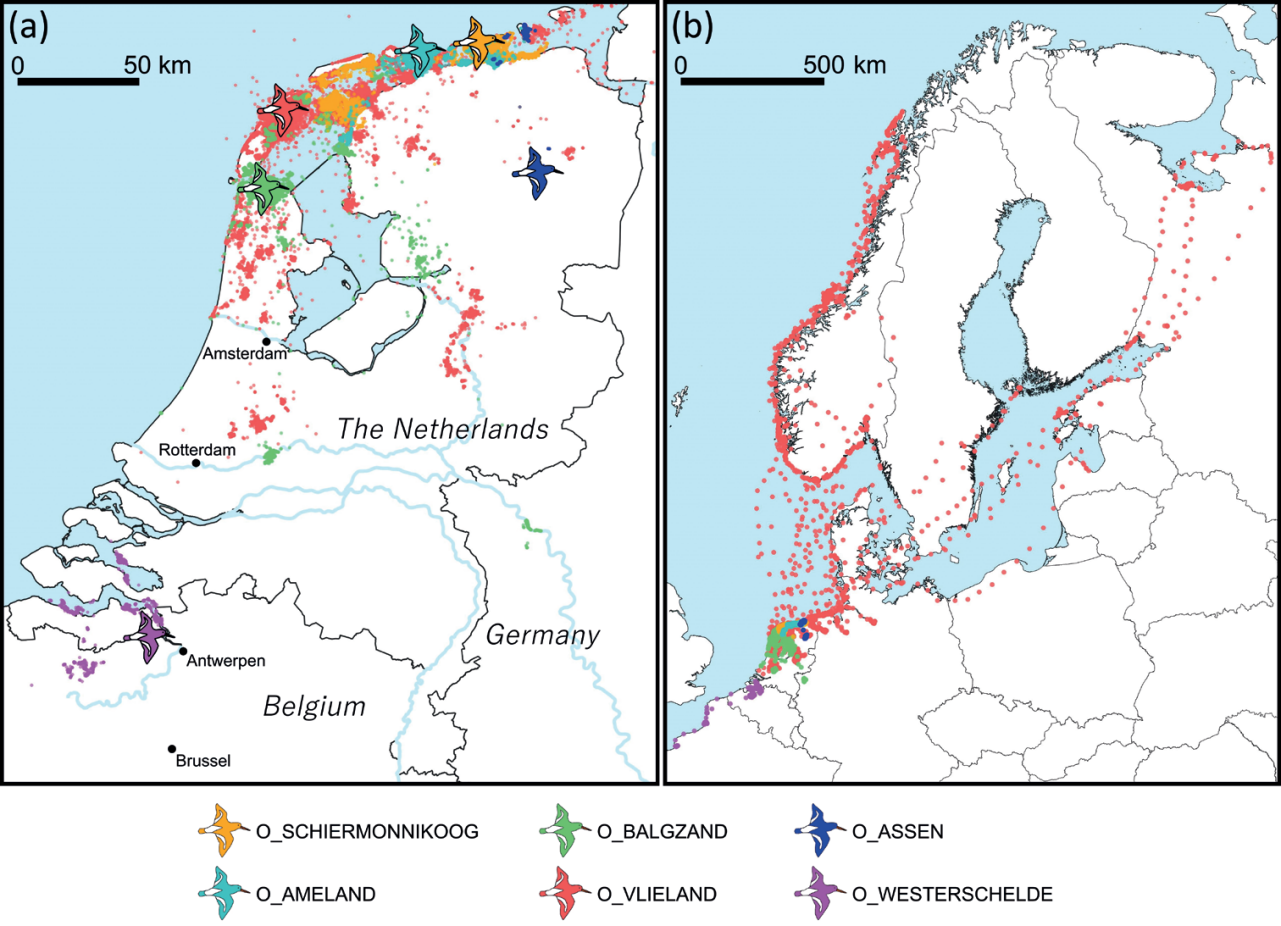
Maps of GPS positions collected from the six different datasets **a** map of the Netherlands and bordering areas of Belgium and Germany showing locations of study sites (indicated by bird symbols) and GPS locations **b** map of northwest Europe showing the full extent of the GPS locations. Maps show GPS locations with hourly intervals; higher frequencies in between GPS locations are omitted in this visualisation.

The datasets contain data from breeding and wintering individuals. A total of 98 breeding individuals were tagged on the Dutch Wadden islands, specifically on the saltmarshes of Schiermonnikoog (O_SCHIERMONNIKOOG), the polder meadows on Ameland (O_AMELAND) and on sandflats on Vlieland (O_VLIELAND). A total of 104 wintering individuals were tagged in the Dutch Wadden Sea at Balgzand (O_BALGZAND) and on sandflats on Vlieland (O_VLIELAND). Inland populations were studied in the Dutch city Assen (O_ASSEN) and in urban and agricultural areas near Antwerp in Belgium (O_WESTERSCHELDE) (Table 1; Fig. 2a). The dataset O_BALGZAND also contains one bird (animal-id: 5331220) that was captured on the nest on the saltmarsh of Schiermonnikoog. The number of tracked individuals per dataset can be found in Table 1. Since many oystercatchers migrate between their breeding and wintering sites, the data coverage extends beyond the sites where birds were tagged. Specifically, the breeding sites of tagged individuals ranged from Antwerp to Scandinavia and Russia, and wintering sites spanned from northern France to the Dutch Wadden Sea (Fig. 2b).

**Table 1.** Dataset characteristics. **Coordinates** are the median coordinates of the catching locations of birds per project; **Individuals** indicates the number of birds that was equipped with a GPS tracker; **Individuals >100 records** indicates the number of individuals for which at least 100 GPS records are available; **GPS records** the total number of GPS positions; **ACC records** indicates the number of accelerometer measurements; **Classified behaviour records** indicate the number of classified behaviours, derived from accelerometer samples (i.e., bursts of consecutive ACC measurements).

	O_SCHIERMONNIKOOG	O_AMELAND	O_BALGZAND	O_VLIELAND	O_ASSEN	O_WESTERSCHELDE
**Title**	Eurasian oystercatchers (*Haematopusostralegus*, Haematopodidae) breeding on Schiermonnikoog (the Netherlands)	Eurasian oystercatchers (*Haematopusostralegus*, Haematopodidae) breeding on Ameland (the Netherlands)	Eurasian oystercatchers (Haematopusostralegus, Haematopodidae) wintering in Balgzand (the Netherlands)	Eurasian oystercatchers (*Haematopusostralegus*, Haematopodidae) breeding and wintering on Vlieland (the Netherlands)	Eurasian oystercatchers (*Haematopusostralegus*, Haematopodidae) breeding in Assen (the Netherlands)	Eurasian oystercatchers (*Haematopusostralegus*, Haematopodidae) breeding in East Flanders (Belgium)
**Movebank study ID**	1605799506	1605803389	1605798640	1605802367	1605797471	1099562810
**First publication date**	2022-01-02	2022-01-17	2022-01-19	2022-01-21	2022-01-17	2022-01-19
**DOI of version described in this paper**	https://doi.org/10.5281/zenodo.6603183	https://doi.org/10.5281/zenodo.6656937	https://doi.org/10.5281/zenodo.6603023	https://doi.org/10.5281/zenodo.5653891	https://doi.org/10.5281/zenodo.5653311	https://doi.org/10.5281/zenodo.5879096
**DOI for all versions**	https://doi.org/10.5281/zenodo.5653477	https://doi.org/10.5281/zenodo.5647596	https://doi.org/10.5281/zenodo.5653441	https://doi.org/10.5281/zenodo.5653890	https://doi.org/10.5281/zenodo.5653310	https://doi.org/10.5281/zenodo.3734898
**Dataset on GBIF**	https://www.gbif.org/dataset/361adb42-c1ea-46ed-979c-281ef027cf8f	https://www.gbif.org/dataset/a700359e-a4fa-47d2-9bca-0b8500528cea	https://www.gbif.org/dataset/833c03c5-fc23-4e77-8689-4e97fcce96f0	https://www.gbif.org/dataset/cd15902d-3ded-41c2-893d-8840e146cbb3	https://www.gbif.org/dataset/226421f2-1d29-4950-901c-aba9d0e8f2bc	https://www.gbif.org/dataset/20bbd36e-d1a1-4169-8663-59feaa2641c0
**Dataset on OBIS**	https://obis.org/dataset/01dbc62a-e166-4752-8547-6db4542ec039	https://obis.org/dataset/3b1da04e-7b8d-4080-ba17-d29909d6d95b	https://obis.org/dataset/2c6aa97e-e886-4564-a55a-48e2e506f014	https://obis.org/dataset/c633b0f8-90bb-43f2-8680-65ac26dd8400	https://obis.org/dataset/550b4cc1-c40d-4070-a0cb-26e010eca9d4	https://obis.org/dataset/132cfd6e-097d-4ee4-b737-58a596dcbe27
**Coordinates**	53.478°N, 6.209°E	53.447°N, 5.823°E	52.943°N, 4.856°E	53.248°N, 4.964°E	53.001°N, 6.570°E	51.275°N, 4.205°E
**Individuals**	43	15	22	103	6	13
**Individuals >100 records**	39	14	20	88	4	7
**GPS records**	602,396	216,111	165,897	4,829,950	20,156	73,047
**First GPS record**	2008-05-31	2010-05-31	2010-06-18	2016-12-02	2018-05-04	2018-05-24
**Last GPS record**	2014-09-02	2013-06-10	2014-04-23	2021-09-06	2019-05-25	2020-04-11
**Outliers**	16	3	6	1,051	4	0
**ACC records**	23,157,229	9,314,045	6,266,870	123,034,944	221,802	1,688,085
**Classified behaviour records**				6,977,784		

### ﻿Temporal coverage

**Figure 3.**
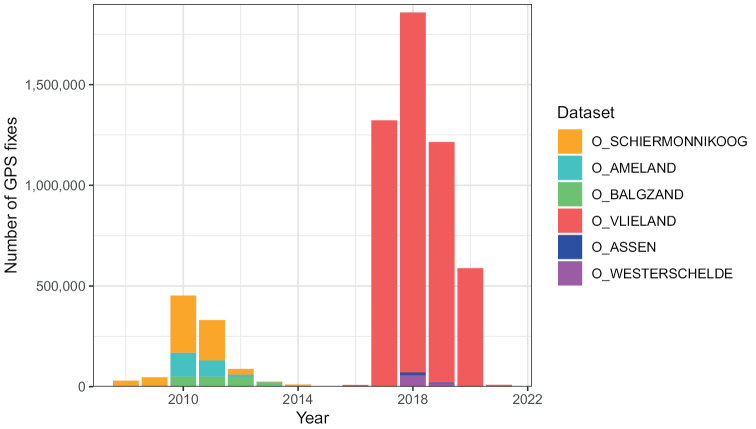
Number of GPS records per dataset per year.

The datasets collectively cover a time period from 2008 until 2021 (Table 1, Fig. 3).

## ﻿Methodology

### ﻿Study extent

Oystercatchers were trapped either in summer on the nest or in winter on their feeding grounds and roost sites. Oystercatchers in breeding populations were always adults that were caught on the nest using walk-in cages. Oystercatchers in wintering populations were caught using mistnets at night, either at low tide (O_BALGZAND) or at high tide (O_VLIELAND). The age of captured birds in winter was classified as either juvenile (1^st^ winter), subadult (2^nd^ winter) or adult (>2^nd^ winter) based on morphology (Cramp et al. 1983). At study sites on Schiermonnikoog and Vlieland, the sex of most birds was determined by DNA analysis of a small blood sample taken from the wing vein and, if available, sex is included in the datasets. Biometrics of trapped birds were taken and included in the datasets, including wing length, tarsus-toe length, bill length, bill tip height, bill tip width (all in mm) and bill tip shape (B = chisel-shaped, H = blunt or hammer-shaped, P = pointed, combined letters indicate intermediate bill tip shapes; van de Pol et al. 2009). Body mass of trapped birds was measured at all study sites and provided for all birds. All birds were equipped with colour rings and with an UvA-BiTS GPS-tracker (Bouten et al. 2013), attached on the back with a harness of Teflon tape that looped around the neck and wings.

A total of 202 individuals were equipped with a GPS tracker (Table 1), but for several individuals no data were registered, possibly due to the tracker malfunctioning. A minimum of 100 GPS records are available for 172 birds. Information on the end of a tracking session is included in the datasets when malfunctioning trackers were removed or when birds were found dead. In the project O_ASSEN, on one bird (animal-id: 5515867) a malfunctioning tracker was replaced by a new tracker.

### ﻿Sampling methodology

The UvA-BiTS (Bouten et al. 2013) trackers used for these studies were all solar powered and had a weight of 18.0 g (O_SCHIERMONNIKOOG), 15.0 g (O_SCHIERMONNIKOOG, O_AMELAND, O_BALGZAND) or 13.5 g (O_SCHIERMONNIKOOG, O_VLIELAND, O_ASSEN, O_WESTERSCHELDE). The trackers record 3D GPS positions and include a tri-axial accelerometer that measures surge X, sway Y and heave Z. Accelerometer measurements were collected in samples of up to 10 s with a frequency of 20 Hz (i.e., a 2.00 s sample consists of 40 consecutive accelerometer measurements; Bouten et al. 2013).

All data collected by the GPS trackers were stored in the internal memory. The data were transmitted remotely to a base station, sometimes via in-between relay stations (Fig. 1c–d). A network of base and relay stations was set up around nesting sites (during the breeding season) or covering high tide roosts (during the non-breeding season). Mobile base stations were occasionally used to download data of birds that were found by colour-ring sightings and resided outside the station network. Data that were downloaded were automatically removed from trackers, thereby freeing up storage for new data. Due to the design of the GPS tracking system, no data could be downloaded from birds that left the study areas with station networks and never returned, except when birds were located based on colour-ring sightings for mobile download, or when trackers were retrieved from dead birds and data was subsequently downloaded from the tracker.

The settings of the GPS trackers, i.e., the intervals between successive GPS fixes, intervals between successive accelerometer samples and length of accelerometer samples, were flexible and could be changed anytime a GPS tracker connected to a base station. Accelerometer samples could follow directly upon a GPS fix or be taken in between GPS fixes. Different settings were used in different seasons and projects. In general, more data were collected when the memory of GPS trackers was empty, i.e., when birds resided within the area covered by receiving stations and data were frequently transmitted to base stations, and when the battery of the GPS trackers was fully charged, i.e., in summer when there is more sunlight. In winter, the battery of the trackers often drained, pausing data collection and consequently, there were data gaps for many birds each winter from November to January. When trackers were collecting data, GPS fixes were recorded at least once per hour, and often at higher frequencies (i.e., every 5, 10 or 15 mins). Sometimes, GPS trackers were set to record bursts with high frequency GPS fixes (i.e., every 16 s) for one or two hours per day during daytime. A total of 6.0 million GPS fixes were collected between 2008 and 2021 (Fig. 3). Data received by the base stations were automatically extracted, post-processed, and stored in a central PostgreSQL database which is part of UvA-BiTS, and accessible to participating researchers only.

**Figure 4.**
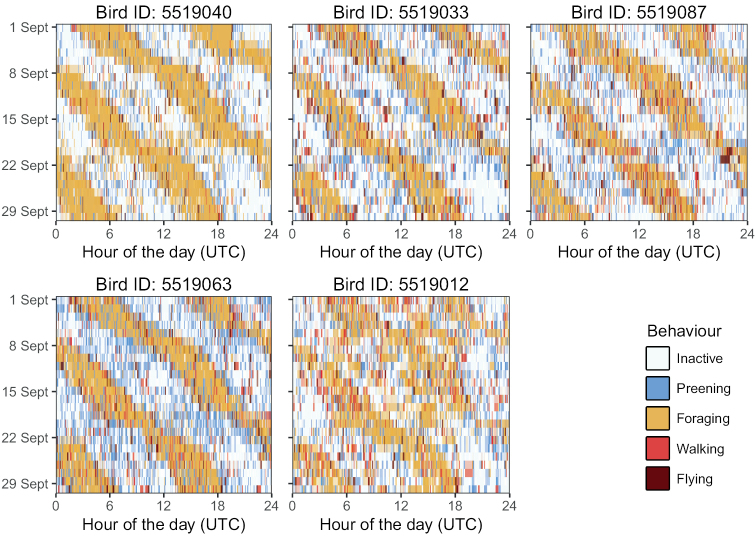
Example of how behavioural classifications included in dataset O_VLIELAND can be used to study time budgets. Time budgets are shown for five individuals in September 2018, which is the non-breeding season when birds were present in intertidal areas on or near Vlieland. The time when birds are foraging shifts every day by 0.5-1.0 hours, as the moments of low tide shifts with the lunar cycle.

The accelerometer samples (i.e., a burst of consecutive accelerometer measurements) can be used to derive movement and behaviour. Typically, behaviour was classified based on summary characteristics (e.g., mean X, standard deviation of Z, etc.) of the accelerometer samples, using a machine learning program that was calibrated with a training dataset. Within these projects, training datasets were acquired by annotating accelerometer samples based on detailed field observations (Shamoun-Baranes et al. 2012) or based on videos that were taken from birds with GPS trackers (van der Kolk et al. 2020a). For O_VLIELAND, a Random Forest model was trained to distinguish five behaviours (flying, walking, foraging, preening, and inactive) and had a prediction accuracy of 94.6% (van der Kolk et al. 2020a). A total of 7.0 million behavioural classifications based on the random forest model were included in the dataset O_VLIELAND, enabling the study of individual variation in behaviour and time budgets (Fig. 4). Note that the classification models were based on annotated behavioural data obtained in intertidal areas mainly in the non-breeding season, and that some behaviours are therefore not distinguished (e.g., no territorial display behaviour was included and incubating behaviour was grouped with inactive behaviour).

### ﻿Quality control

GPS fixes that were likely incorrect (i.e., outliers) were marked in two ways: manually by the researcher in the UvA-BiTS database (indicated as TRUE in *manually-marked-outlier*) and automatically (in https://github.com/inbo/bird-tracking) before uploading to Movebank for GPS-fixes with speed above 45 ms^-1^ or GPS-fixes with an angle below 30° and speed above 15 ms^-1^ (indicated as TRUE in *import-marked-outlier*). The outlier count for each study is provided in Table 1. The rationale for these criteria is that migrating oystercatchers can travel at speeds up to around 30 ms^-1^ but then move in a more or less straight direction (translating into a large angle). Outliers are typically characterised by high speed and a sharp angle (i.e., a movement towards the outlier is followed by a movement back to the original location). These criteria target mostly the largest outliers, and depending on the goal of data use, stricter filtering criteria may need to be applied.

### ﻿Data publication

To make the data openly available, all data were uploaded to Movebank (https://www.movebank.org), an online platform and database specialized in storing animal tracking and bio-logging data. The Movebank data model enables the description of animals, tags, deployments, detections, and other measurements recorded by or derived from animal-borne sensors, such as acceleration data (Kays et al. 2022). For the six datasets, reference data containing information about the animals, tags and deployments, as well as GPS and acceleration data were downloaded from the UvA-BiTS database and transformed to the Movebank data format (Movebank 2021) using SQL queries and R scripts (https://github.com/inbo/bird-tracking). This guarantees a consistent approach for all datasets and allows for repeating the process when new data become available for active studies. These data (and for O_VLIELAND additional behavioural classifications) were then uploaded to the Movebank database, with one study-identifier for each dataset (Table 1), documented with metadata and made available under an open Creative Commons Zero waiver.

To enable long-term and low-tech data preservation, data were also deposited as CSV files on the research repository Zenodo (https://zenodo.org). GPS, acceleration and behavioural data were split into separate files per year and compressed, making it easier to download data in manageable chunks. A datapackage.json file was included for each deposit, making it a Frictionless Data Package (https://specs.frictionlessdata.io/data-package/), a simple container format for tabular data. This file references all CSV files, organizes them into resources (reference-data, gps, acceleration, and biometric-measurements) and describes each of their fields, including data type, format and definition according to the Movebank Attribute Dictionary (Movebank 2021). The datapackage.json file also facilitates programmatic access to the dataset, such as with the R package ‘frictionless’ (Desmet and Oldoni 2022). Each deposited version on Zenodo is assigned a DOI upon publication, as well as a versionless DOI that always points to the latest version of the deposit (see Table 1 for their Zenodo identifiers).

Movement data can be used as general-purpose occurrence data. To enable wider discoverability and use, we reformatted our datasets to incorporate them in the Global Biodiversity Information Facility (GBIF, https://www.gbif.org) and the Ocean Biodiversity Information System (OBIS, https://obis.org). Reference and GPS data (excluding outliers; including fields informing on accuracy, e.g., *coordinateUncertaintyInMeters*) were transformed to Darwin Core (Wieczorek et al. 2012), and down-sampled to the first record per hour, to not needlessly flood GBIF and OBIS with high-frequency movement data. Metadata were transformed to the Ecological Metadata Language (EML) and included the same authors, keywords and DOI, and explained that data are down-sampled. The transformation process to Darwin Core and EML was automated with the custom developed R package ‘movepub’ (Desmet 2022). This automated approach includes fields that are not available for the datasets described in this study (*organismName* and *reproductiveCondition*), but could be included for future datasets that are transferred using this process. The datasets on Zenodo, GBIF and OBIS cross reference each other as well as the datasets on Movebank.

### ﻿Method steps

#### Sampling

1. Researcher defines a GPS tracker measurement scheme, which could be updated anytime GPS trackers were connected to a base station.
2. Researcher captures bird, takes biometrics, attaches UvA-BiTS GPS tracker, and releases bird.
3. Researcher records or updates metadata about bird, GPS tracker and deployment in UvA-BiTS database.
4. GPS tracker records data.
5. GPS tracker automatically transmits recorded data when connected with a base station.
6. Recorded data were automatically extracted, post-processed, and stored in the central PostgreSQL database of UvA-BiTS.
7. Data stream stops when a bird no longer returns to the study area, if a bird dies, if a GPS tracker malfunctions or if receiver stations are removed.

#### Data publication

1. Data (reference, GPS and acceleration) were exported from UvA-BiTS in the Movebank data format.
2. GPS outliers were marked.
3. Data were uploaded to the appropriate study on Movebank and made publicly available.
4. Data were exported from Movebank and archived on Zenodo as a Frictionless Data Package, where each update has a version with a DOI.
5. Data were downsampled to one location per hour and formatted as Darwin Core, allowing exports to GBIF and OBIS.

## ﻿Additional information

The following information is not included in the datasets and is available upon request: (1) resightings of tagged birds based on colour-ring observations by volunteers; and (2) manually annotated accelerometer data and a classification model to classify behaviour based on accelerometer samples following van der Kolk et al. (2020a), or classifications for specific time periods and datasets that are not already included.

## ﻿Related publications

The described datasets were used in the following publications: O_SCHIERMONNIKOOG (Shamoun-Baranes et al. 2012; Ens et al. 2014b; Bakker et al. 2021), O_AMELAND (Ens et al. 2014b; Bakker et al. 2021), O_BALGZAND (Dokter et al. 2017; Bakker et al. 2021), O_VLIELAND (Linssen et al. 2019; van der Kolk et al. 2020a, b, 2021a, b, 2022; Bakker et al. 2021; van der Kolk 2021), O_ASSEN (Dijkstra and Dillerop 2018, 2019), O_WESTERSCHELDE (Vanoverbeke et al. 2020).

## References

[B1] Allen A Ens BJ van de Pol M Frauendorf M van der Kolk HJ de Kroon H Jongejans E (2018) Cumulative Human Impacts on biRd Populations (CHIRP): A multi-tiered approach to conserving the near-threatened Eurasian Oystercatcher. 5^th^ European Congress of Conservation Biology. 10.17011/conference/eccb2018/107685

[B2] Allen AM Jongejans E van de Pol M Ens BJ Frauendorf M van der Sluijs M de Kroon H (2022) The demographic causes of population change vary across four decades in a long‐lived shorebird. Ecology 103(4): e3615. 10.1002/ecy.3615 PMC9286424 34921394

[B3] Bakker W Ens BJ Dokter AM van der Kolk HJ Rappoldt K van de Pol M Troost K van der Veer HW Bijleveld AI van der Meer J Oosterbeek K Jongejans E Allen AM (2021) Connecting foraging and roosting areas reveals how food stocks explain shorebird numbers. Estuarine, Coastal and Shelf Science 259: 107458. 10.1016/j.ecss.2021.107458

[B4] Bouten W Baaij EW Shamoun-Baranes J Camphuysen KC (2013) A flexible GPS tracking system for studying bird behaviour at multiple scales. Journal of Ornithology 154(2): 571–580. 10.1007/s10336-012-0908-1

[B5] Cramp S Simmons KLE Brooks DC Collar NJ Dunn E Gillmor R Hollom PAD Hudson R Nicholson EM Ogilvie MA Olney P Roselaar C Voous KH Wallace D Wattel J Wilson M (1983) Handbook of the birds of Europe, the Middle East and North Africa. The birds of the Western Palearctic: 3. Waders to gulls. Oxford University Press, Oxford.

[B6] Desmet P (2022) movepub: Prepare Movebank data for publication. R package version 0.1.0. https://github.com/inbo/movepub

[B7] Desmet P Oldoni D (2022) frictionless: Read and Write Frictionless Data Packages. R package version 0.10.0. 10.5281/zenodo.5815355

[B8] Dijkstra B Dillerop R (2018) Eerste bevindingen zenderonderzoek Scholeksters *Haematopusostralegus* in Assen. Drentse vogels 32: 39–52.

[B9] Dijkstra B Dillerop R (2019) Over de reis van twee Drentse Scholeksters *Haematopusostralegus* naar hun overwinteringsgebied in de Waddenzee. Drentse vogels 33: 30–35.

[B10] Dokter AM van Loon EE Rappoldt C Oosterbeek K Baptist MJ Bouten W Ens BJ (2017) Balancing food and density‐dependence in the spatial distribution of an interference‐prone forager. Oikos 126(8): 1184–1196. 10.1111/oik.04139

[B11] Ens BJ van de Pol M Goss-Custard JD (2014a) Chapter Eight - The Study of Career Decisions: Oystercatchers as Social Prisoners In: Naguib M Barrett L Brockmann HJ Healy S Mitani JC Roper TJ Simmons LW (Eds) Advances in the study of behavior.Academic Press, Vol 46, 343–420. 10.1016/B978-0-12-800286-5.00008-0

[B12] Ens BJ Bom RA Dokter AM Oosterbeek K de Jong J Bouten W (2014b) Nieuwe ontdekkingen en mogelijkheden in het onderzoek aan Scholeksters dankzij het UvA Bird Tracking Systeem. Limosa 87(2–3): 117–128.

[B13] Goss-Custard JD (1996) The Oystercatcher. From Individuals to Populations. Oxford University Press, Oxford.

[B14] Kays R Davidson SC Berger M Bohrer G Fiedler W Flack A Hirt J Hahn C Gauggel D Russell B Kölzsch A Lohr A Partecke J Quetting M Safi K Scharf A Schneider G Lang I Schaeuffelhut F Landwehr M Storhas M van Schalkwyk L Vinciguerra C Weinzierl R Wikelski M (2022) The Movebank system for studying global animal movement and demography. Methods in Ecology and Evolution 13(2): 419–431. 10.1111/2041-210X.13767

[B15] Linssen H van de Pol M Allen AM Jans M Ens BJ Krijgsveld KL Frauendorf M Van der Kolk HJ (2019) Disturbance increases high tide travel distance of a roosting shorebird but only marginally affects daily energy expenditure. Avian Research 10(1): 1–11. 10.1186/s40657-019-0171-8

[B16] Movebank (2021) Movebank attribute dictionary. World Wide Web electronic publication. http://vocab.nerc.ac.uk/collection/MVB/current/ [accessed in January 2022]

[B17] Shamoun-Baranes J Bom R van Loon EE Ens BJ Oosterbeek K Bouten W (2012) From sensor data to animal behaviour: An oystercatcher example. PLoS ONE 7(5): e37997. 10.1371/journal.pone.0037997 PMC3365100 22693586

[B18] Stillman RA Goss‐Custard JD (2010) Individual‐based ecology of coastal birds. Biological Reviews of the Cambridge Philosophical Society 85(3): 413–434. 10.1111/j.1469-185X.2009.00106.x 19961470

[B19] van de Pol M Ens BJ Oosterbeek K Brouwer L Verhulst S Tinbergen JM Rutten AL Jong MD (2009) Oystercatchers’ bill shapes as a proxy for diet specialisation: More differentiation than meets the eye. Ardea 97(3): 335–347. 10.5253/078.097.0309

[B20] van de Pol M Atkinson P Blew J Crowe O Delany S Duriez O Ens BJ Hälterlein B Hötker H Laursen K Oosterbeek K Petersen A Thorup O Tjørve K Triplet P Yésou P (2014) A global assessment of the conservation status of the nominate subspecies of Eurasian Oystercatcher *Haematopusostralegusostralegus*. International Wader Studies 20: 47–61.

[B21] van der Kolk H (2021) Stay or fly away? Impact of human disturbance on shorebird individuals and populations. PhD Thesis. Radboud University, Nijmegen.

[B22] van der Kolk H Ens BJ Oosterbeek K Bouten W Allen AM Frauendorf M Lameris TK Oosterbeek T Deuzeman S de Vries K Jongejans E van de Pol M (2020a) Shorebird feeding specialists differ in how environmental conditions alter their foraging time. Behavioral Ecology 31(2): 371–382. 10.1093/beheco/arz189

[B23] van der Kolk H Allen AM Ens BJ Oosterbeek K Jongejans E van de Pol M (2020b) Spatiotemporal variation in disturbance impacts derived from simultaneous tracking of aircraft and shorebirds. Journal of Applied Ecology 57(12): 2406–2418. 10.1111/1365-2664.13742

[B24] van der Kolk H Ens BJ Frauendorf M Jongejans E Oosterbeek K Bouten W van de Pol M (2021a) Why time‐limited individuals can make populations more vulnerable to disturbance. Oikos 130(4): 637–651. 10.1111/oik.08031

[B25] van der Kolk H Ens BJ Jongejans E Frauendorf M Allen AM de Kroon H van de Pol M (2021b) Conclusies uit vier jaar onderzoek naar vliegtuigverstoring van Scholeksters (*Haematopusostralegus*) op Vlieland. Twirre 31(2): 9–18.

[B26] van der Kolk H Ens BJ Oosterbeek K Jongejans E van de Pol M (2022) The hidden cost of disturbance: Eurasian Oystercatchers (*Haematopusostralegus*) avoid a disturbed roost site during the tourist season. The Ibis 164(2): 437–450. 10.1111/ibi.13035

[B27] Vanoverbeke J Spanoghe G De Regge N Van Ryckegem G (2020) Foerageergedrag van scholeksters op de Westerschelde. Rapporten van het Instituut voor Natuur- en Bosonderzoek: 23. 10.21436/inbor.18345084

[B28] Wieczorek J Bloom D Guralnick R Blum S Döring M Giovanni R Robertson T Vieglais D (2012) Darwin Core: An evolving community-developed biodiversity data standard. PLoS ONE 7(1): e29715. 10.1371/journal.pone.0029715 PMC3253084 22238640

